# Analysis of Flow Characteristics between Tandem Flexible Structures Based on PIV: Substantial Applications for the Removal of Microplastics

**DOI:** 10.3390/mi15010100

**Published:** 2024-01-04

**Authors:** Hyeonjin Lee, Bongliba T. Sangtam, Heejoong Seong, Jeong Jae Kim, Hanwook Park

**Affiliations:** 1 Department of Biomedical Engineering, Soonchunhyang University, 22 Soonchunhyang-Ro, Asan 31538, Chungnam, Republic of Korea; leeha@sch.ac.kr (H.L.); drsangtam@sch.ac.kr (B.T.S.); yrsung718@naver.com (H.S.); 2 Department of Mechanical Engineering, Hanbat National University, 125, Dongseo-daero, Yuseong-gu, Daejeon 34158, Republic of Korea

**Keywords:** Particle image velocimetry, flexible structure, tandem arrangement, microplastics

## Abstract

This study emphasizes the potential risk posed by microplastics, particularly in tap water. Numerous studies have reported the removal of microplastics, but the limitations in addressing this issue remain challenging. To tackle this problem, a new method is introduced using tandem flexible structures (FSs) for microplastic removal. The present study focused on understanding the hydrodynamic characteristics between FSs to utilize microplastic removal. This comprehension of fluid flow and FSs offers valuable insights for improving the efficiency of microplastic removal methods. Therefore, the optimal conditions for removing microplastics were experimentally investigated inside the FSs gap region. Based on the gap distance and height, the flow structures between FSs were investigated. A small secondary vortex structure that could trap particles from upstream was continuously maintained behind the upstream FSs under certain geometric conditions. It is shown that this vortex structure has an effective way of confining the particles from upstream. The persistency of a small secondary vortex was also evaluated. This study may be helpful to researchers working on microplastic removal and FSs with a tandem arrangement.

## 1. Introduction

Plastic has become an integral aspect of human life, ranging from its use in shopping to numerous household products. As a result, many waste products found in the ocean contain plastic. Transitioning to greener energy sources is important for advancing energy management and ensuring a more sustainable and environmentally friendly future [1,2]. Microplastics are plastics with dimensions of ≤5 mm, and they have a variety of shapes and sizes [3]. Based on the study reported by ‘Plastic Europe’, the worldwide plastic production in 2021 was 390.7 Metric ton, with nearly 90.2% contributed from fossil sources [4]. There are currently 20 distinct microplastic particles present in the environment, the majority of which are polyethylene (PE), polypropylene (PP), polystyrene (PS), and polyvinyl chloride (PVC) [5]. In wastewater, these microplastics present in a very tiny range of ≤600 µm, and are often encountered in treated plants and are difficult to remove using conventional techniques [6]. These materials are not easy to degrade, and they can persist for many years in the environment, and potentially over centuries. Throughout the gradual decomposition process, larger plastic items are likely to break down into meso-plastics (ranging from 5 to 40 mm), microplastics (particles measuring 1–5000 μm), and nano-plastics (particles at or below 0.1 μm) before ultimately undergoing complete decomposition [7,8]. These microplastics are found in water bodies such as rivers, seas, and lakes. When plastic degrades through sunlight and rain, leftover particles may find their way through the soil and into the groundwater surface [9]. Humans are exposed to these tiny particles through ingestion, inhaling, and skin contact [10]. The critical origin of microplastics in an aquatic system includes tiers, laundry, household dust, synthetic fabrics, and sewage treatment [11,12,13]. Microplastics are sometimes referred to as “the silent menace” since their small size makes them difficult to detect [14]. As a result of microplastics pervasiveness in the environment, many living organisms have consumed these tiny plastics through various sources. Microplastics are one of the most widespread and continual kinds of pollution generated by humans on earth. It is estimated that 5.25 trillion particles of plastic are floating on the surface of the ocean, out of which 92% are microplastics with a size less than 5 mm [15,16,17]. Microplastic fragments measuring less than 5 mm abound in the environment, spreading, migrating, and accumulating in natural ecosystems worldwide, throughout the poles to the equator, and from the ocean’s surface to the seafloor. Furthermore, this debris can be found on both urban beaches and undisturbed sediments [17,18]. This common and constant form of pollution shows an ongoing and open threat to marine life in oceans. Water is the major transporter of microplastics, and wastewater plants collect millions of microplastics daily via the urban sewage system. As these microscopic contaminants are not fully removed from wastewater treatment operations, a substantial quantity of microplastics is discharged into water bodies [19]. Several studies, however, show the existence of microplastics in drinking water treatment plants, which eventually permeate home tap water [20]. Generally, household pipes are made of PVC materials, and after they have been used for a certain period may degrade, which potentially could contribute to microplastics in tap water [21]. Over 81% of 159 samples that were collected from 14 different countries were tested to check the presence of microplastics [22]. The study also used 11 different brands of 259 different water bottles from nine countries that were analyzed for microplastic presence in these bottles [23]. They observed that 93% of these bottled waters contained microplastic, with an average of 10.4 microplastic particles with sizes larger than 100 µm/L of bottled water, comprising smaller particles ranging from 6.5 to 100 µm, with 325 average microplastic particles/L in bottled water. Tong et al. [24] collected 38 different tap water samples from various urban areas in China and analyzed the microplastic presence. They found that the presence of microplastics ranged from 440 ± 275 piece/L, with the most common size range below 50 µm. Microplastics in tap water come in several shapes, such as particles, pieces, fibers, and sprays, and most of them include polyethylene (PE) and polypropylene (PP) [25]. As a result, it is essential to acquire microplastic-free tap water. Many filters have been designed to eliminate tiny particulates in the flow, but the filters have drawbacks such as being expensive, noisy, and having a considerable pressure drop [26]. Therefore, alternative methods are required to remove micromaterials, such as microplastics and particulate matter. 

Understanding hydrodynamic characteristics, particularly fluid flow, is essential for the effective design of a microplastic removal system. Sun et al. [27] studied the fluid dynamics interaction of turbulence and chemical reaction. One of the advanced methods for measuring fluid flow visualization is the Particle Image Velocimetry (PIV) technique. It is non-intrusive and can be used for measuring the velocity distributions in fluid flow [28]. Kitagawa et al. [29] reported microplastic particle tapping in microfluid devices using various shaped pillars. Using the finite element simulation, the intricate movement of trapping microspheres was studied via the particle trap array device [30]. A similar mechanism of particle trapping is exhibited in animal nose hairs by blocking the inhalation of harmful materials from the outside [31]. These nasal hairs are made up of several flexible structures (FSs), which help to remove dust. Nasal hairs protect the respiratory tract from various airborne diseases [26]. Also, when compared to rigid structures, the FSs exhibit less pressure drop [32]. Therefore, by constructing FSs that can accurately collect tiny particles by controlling geometric parameters and flow, the particle could potentially eliminate extra suction devices or frequent cleaning. For determining the efficiency of microplastic removal, it is essential to carry out experiments on flow dynamics near FSs. The flow within tandem structures affects complex flow characteristics such as recirculation, and vortex shedding. In the case of a tandem arrangement, the flow from the upstream structures changes the flows for the downstream structure. The current work bridges an existing knowledge gap in microplastic removal by focusing on hydrodynamics characteristics of fluid flow. The study focuses on understanding the flow structure within the FSs, which will aid in understanding the effectiveness of capturing microplastics from the upstream flow via the FSs. The tandem structures generate flow between the structures and the FSs exhibit different flows according to gap distance, structure height, and Reynolds number (Re) [33,34,35]. The PIV technique was used to analyze the FSs within the gap between tandem FSs. This approach is widely used to obtain velocity information near bluff bodies [36,37].

In the present study, tandem FSs are proposed as an alternative method for collecting microplastics. The study focused on hydrodynamics, particularly the formation of a small secondary vortex structure. This flow structure has the advantage of trapping microplastics between the FSs. The effects of gap distance and the height of FSs on the flow structures were also demonstrated. Based on the experimental data, the flow structures and bending performance of FSs were examined, and the flow characteristics were classified into three types. The persistency of the secondary vortex was also exhibited to prevent the washing out of gathered microplastics. The results of the present study would be helpful in the development of new microplastic removal devices.

## 2. Materials and Methods

### 2.1. PIV Experiment

**Figure 1:**
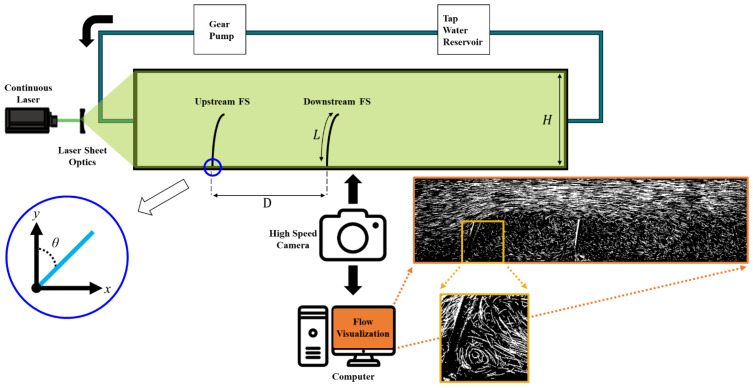
Schematic diagram of FSs set-up.

In the present study, the detailed experimental setup of FSs is shown in Figure 1. The orange box illustrates the visualized results using a streakline, whereas the yellow box depicts the observation of a small secondary vortex behind the upstream flexible structure (FS) using a streakline. The FSs are designed based on the cilia inspired. The flow circuit includes the following basic components: a reservoir for tap water storage, a steady gear pump used for maintaining the uniform liquid flow rate, an experimental model for predicting the fluid dynamics, and circular pipes that direct the fluid movement. The flow behaviors between FSs were studied using a 2D model. An experimental model with a rectangular cross-sectional shape was adopted and used to examine the flow behavior. The model was made of acrylic material, and it was delineated into three parts: as inlet section, the experimental part, and the outlet region. This design enables a change of FSs during the experimentation phase. To maintain a uniform upstream flow condition, a mesh was installed at the inlet section entrance with a length of 30 cm. The Polydimethylsiloxane (PDMS) blocks were incorporated with the experimental duct model and ensured that the cross-sectional dimensions of 50 mm width and 20 mm height remained uniform throughout. FSs were carefully placed across the experimental section to record and analyze flow characteristics. In the experiment, to simulate a water supply pipe two FSs are placed inside the 2D duct, one in the upstream and another along the downstream position. The origin point was determined at the bottom of the upstream FS as (x, y) = (0, 0). To define the position of the FSs in the experimental setting, the bending angle (*θ*) is computed between the y-axis and the tip of the FSs. To prevent water leakage, each component was carefully sealed with a silicon gasket. A curved lens was used to broaden the laser beam and laser sheet illuminated by an acrylic duct to visualize flow movements. The 2W continuous green laser beam (532 nm wavelength, MGL-F-532, Shanghai Dream Laser Technology, Ltd., China) was used in this study, resulting in an illuminated laser sheet. Tap water was used for experimenting and this fluid was supplied from a gear pump (ISMATAC IP65). To replicate tap water flow, a flow rate of 6.45 L/min was set and the corresponding Re of 3440 was maintained. The tracer particle used was silver-coated hollow glass spheres with an average diameter of 44 μm (Potters Industries, USA), and the equivalent particle was chosen based on the microplastic size range. A high-speed camera (Mini-UX 50, Photron, Japan) equipped with a Tokina 100 mm atx-i F2.8 FF MACRO lens was used to record the particle images. The frame rate was kept at 2500 fps and the field of view was 1280 (horizontal) and 296 (vertical) pixels. Images were captured at each 5 s duration. Using a two-frame cross-correlation PIV algorithm, velocity field data were acquired. The instantaneous velocity vectors were obtained by averaging 25 subsequent velocity vectors with a window size of 32 × 32 pixels and 50% overlapping. The Stokes number is a dimensionless quantity that determines the particle behavior in fluid flow. In experimental fluid dynamics, the Stokes number quantifies tracer particles along the flow, especially in the PIV experiment. It also determined how rapidly these small particles travel in the direction of fluid movement, also known as fluid velocity. The accuracy of tracing seeding particles in fluid flow is represented by a Stokes number (Stk) of approximately 6.042 × 10^−4^. The Stk value is less than 0.1, indicating that the tracing accuracy error is below 1% [38]. The following Stokes number equation is expressed as

(1) $$Stk=\frac{u_{c}\tau_{p}}{L_{c}}$$

(2) $$\tau_{p}=\frac{\rho_{p}{\;d_{p}}^{2}}{18{\;\mu }_{f}}$$

where *ρ_p_* is the particle density, *d_p_* is the particle diameter, *μ_f_* is the kinematic fluid viscosity, $L_{c}$ is the characteristic length, and *u_c_* is the characteristic velocity.

### 2.2. Experimental Model

**Figure 2:**
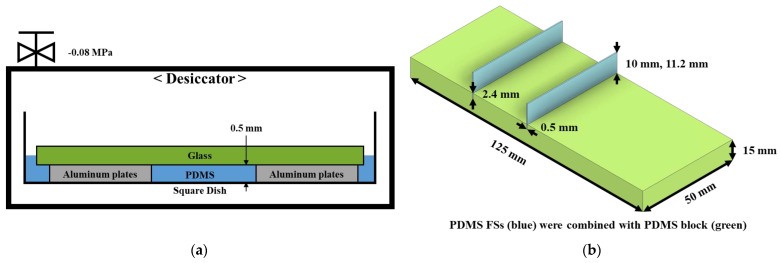
(**a**) Fabrication of FSs with 0.5 mm thickness. (**b**) Geometric parameters of FSs.

The FSs were made of polydimethylsiloxane (PDMS) with a thickness of 0.5 mm. Figure 2a depicts the FSs fabrication process, in which aluminum plates are fastened within square dishes using solid adhesive. Both the aluminum plate and solid adhesive were maintained with a uniform thickness of 0.5 mm. The slide glass was placed on the aluminum plates, and the silicon base and curing agent were added to a square dish. To remove air bubbles from the PDMS solution, the suction pump was switched on to produce a vacuum inside the desiccator. Subsequently, FSs were positioned in a convection oven and maintained at a temperature of 40 °C for 3 h. FSs are placed inside the PDMS block, as shown in Figure 2b. To ensure a stable and solid position, the FSs (blue sheet) were firmly placed into the PDMS block (green) at a depth of 2.4 mm.

Although the constant flow supplied momentum to the FSs, the distance of FSs was maintained throughout the experiments. The FSs model lengths for *L/H* ratios of 0.5 and 0.56 were maintained at 12.4 mm and 13.6 mm, respectively. For *L/H* = 0.5, PDMS FSs measuring 50 mm wide and 12.4 mm tall (original FSs dimensions) were mounted on a PDMS block. To secure the upstream and downstream FSs, this PDMS block was designed with grooves. As the groove depth was kept at 2.4 mm, the FSs inserted into the block height were set to 10 mm. To study various flow structures according to gap distance, experimental models with varying *D/H* ratio values of 0.5, 1.5, and 2.5 were used. Each of these distance ranges was chosen for understanding different flow behaviors. The PDMS block was embedded inside the acrylic duct without changing its cross-sectional area. The Young’s modulus value is estimated by applying the bending amount calculation, as follows:

(3) $$E=\frac{{qL}^{4}}{8I}\times \frac{1}{\delta }$$

where *δ* is the deflection, *q* is the weight per unit length, *L* is the length of the leaflet, *E* is Young’s modulus, and *I* is the area moment of inertia of the cross-section. This is a two-dimensional experiment, and the FSs have a rectangular cross-section. The Young’s modulus value of FSs used in this study was 650 kPa.

## 3. Results and Discussion

### 3.1. Effects of Gap Distance of FSs

**Figure 3:**
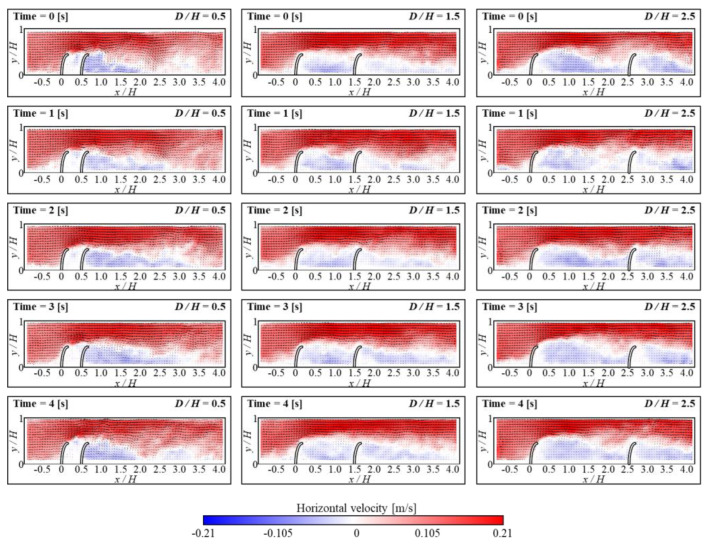
Instantaneous velocity vectors and horizontal velocity (*u*) contours at different times for *D/H* = 0.5 (**left column**), *D/H* = 1.5 (**middle column**), and *D/H* = 2.5 (**right column**).

**Figure 4:**
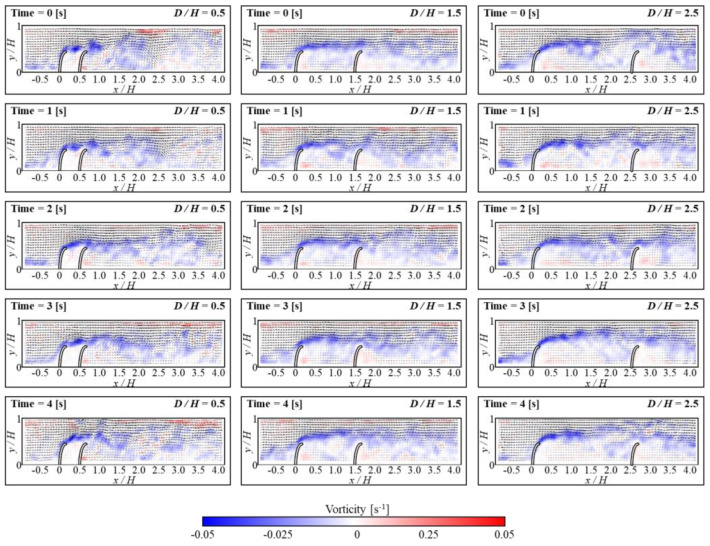
Instantaneous velocity vectors and vorticity contours at different times for *D/H* = 0.5 (**left column**), *D/H* = 1.5 (**middle column**), and *D/H* = 2.5 (**right column**).

Figure 3 and Figure 4 show the temporal evolutions of the horizontal velocity and vorticity contours along with the velocity vectors based on gap distance. The FSs presented in the figures are not original, but were drawn for better understanding. During the experiment, the *L/H* was fixed at 0.5, and the Reynolds number (Re) was maintained at 3440. Based on *D/H* ratios, the flow characteristics inside the gap region were classified into three patterns [39]. At a *D/H* ratio of 0.5, the flow between FSs remained constant, and the flow velocity was approximately zero for all time positions. It was found that the liquid from the upstream remains separated from the inside gap area. Due to the distinct nature of the two flows, the flow inside the gap exhibits extended structure, and the FSs act as a single structure. However, when the *D/H* ratio was 1.5, the reattachment generated by the separated flow from the upstream is appeared near downstream FS. With the vibration generated by the upstream FS, the *x/H* of the reattachment region shifts from 1 to 1.5. As a result, a significant recirculation was developed under the reattachment region, and a small secondary vortex was developed near the bottom of the upstream FS. Throughout the experiments, this secondary vortex was maintained uniformly. The particles from the upstream then combine with the large circulation flow, which continues to flow alongside the small secondary vortex near the bottom of upstream FSs. Based on this study, the formation of vortices in the gap space can be used as an effective mechanism for capturing particles from upstream. When *D/H* was 2.5, a phenomenon called vortex shedding occurred. This happens when multiple vortices are periodically released into the fluid flow. Reattachment happened when *x/H* was between 1 and 2, and at this range, vortex shedding was observed. As a result, significant recirculation zones emerged beneath the reattachment region. In contrast to *D/H* at 1.5, larger recirculation was observed, and it moved towards the upstream FS. This led to a dissipation of the small secondary vortex, which hampered the continuous flow and disrupted particle gathering from upstream.

**Figure 5:**
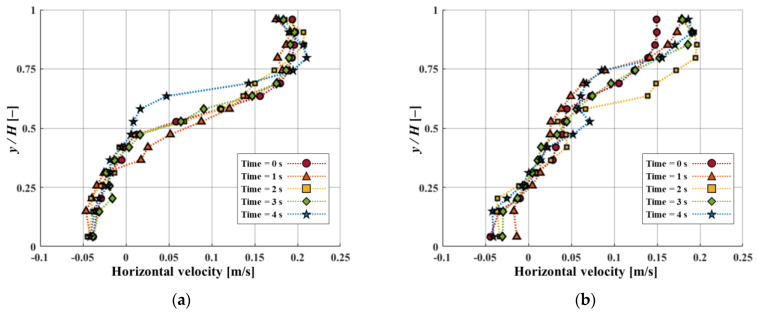
Variations in horizontal velocity along the *y/H* positions for (**a**) *D/H* = 1.5. (**b**) *D/H* = 2.5 with different time intervals.

Figure 5a,b depict horizontal velocity profiles for both *D/H* at 1.5 and 2.5 at different time intervals where *x/D* is 80% positions of *D/H*. A negative horizontal velocity profile showed the presence of recirculation flow in the gap area. At *D/H* = 1.5, negative horizontal velocity occurred when *y/H* was close to 0.4. When *y/H* was less than 0.25, it was found that the horizontal velocity profile at *D/H* = 2.5 was noted as a negative value. This implies that when *D/H* = 1.5, recirculation flow originated in the gap region. Moreover, negative horizontal velocities were consistently maintained with time. As a result, a large recirculation flow was maintained without causing significant changes inside the gap region. When *D/H* is 2.5, the variation in horizontal velocity over time is found to be larger than when *D/H* = 1.5. These significant changes in horizontal velocity result from vortex shedding, leading to a more quantitative indication of the vortex shedding phenomenon.

### 3.2. Effects of Height of FSs

**Figure 6:**
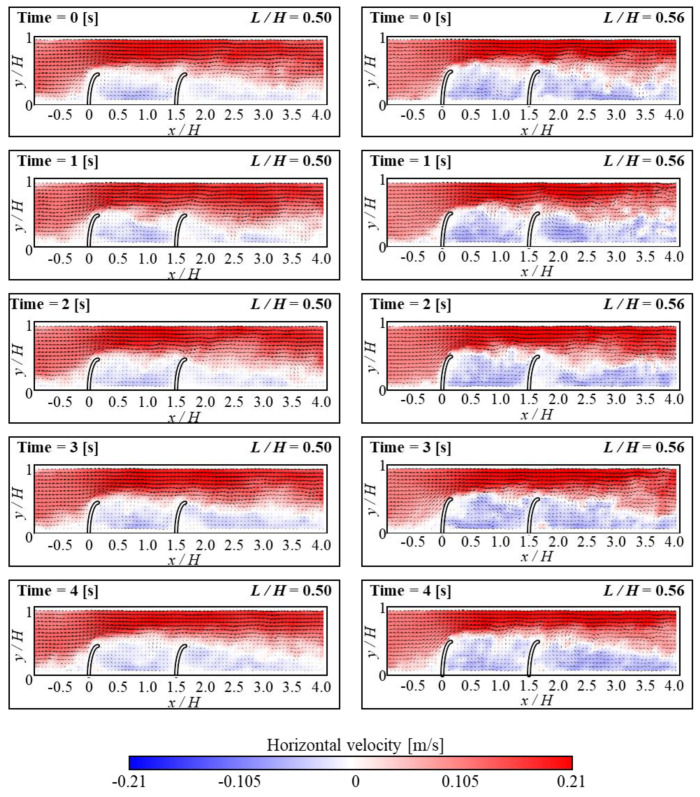
Instantaneous velocity vectors and horizontal velocity (*u*) contours of flow at different time intervals for *L/H* = 0.5 (**left column**) and *L/H* = 0.56 (**right column**).

**Figure 7:**
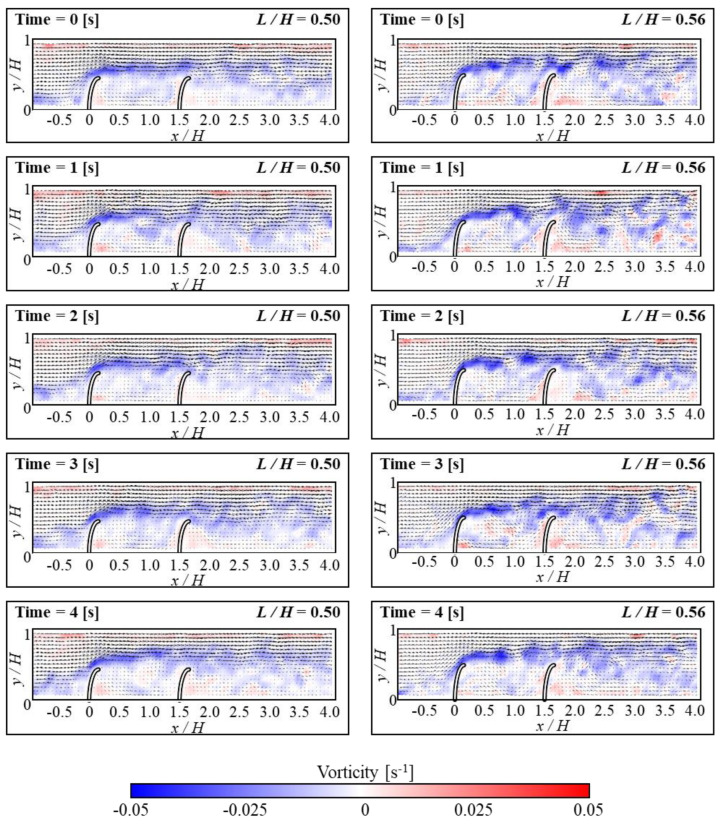
Instantaneous velocity vectors and vorticity contours of flow at different time intervals for *L/H* = 0.5 (**left column**) and *L/H* = 0.56 (**right column**).

**Figure 8:**
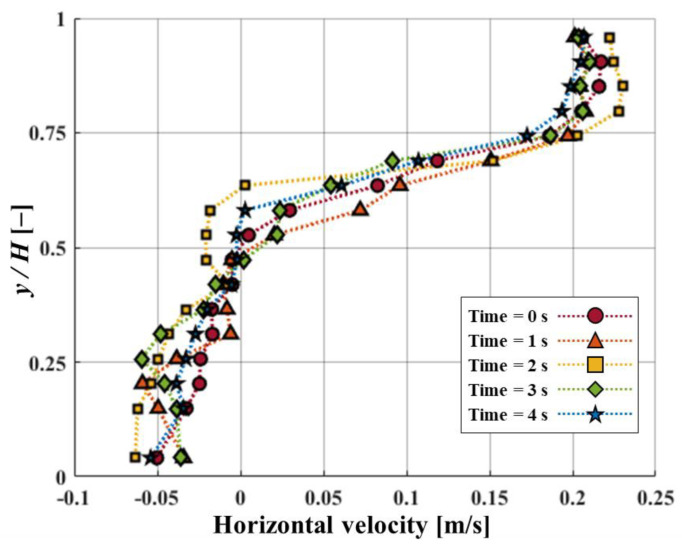
Variations in horizontal velocity along the *y/H* positions at *L/H* = 0.56 with time changes.

**Figure 9:**
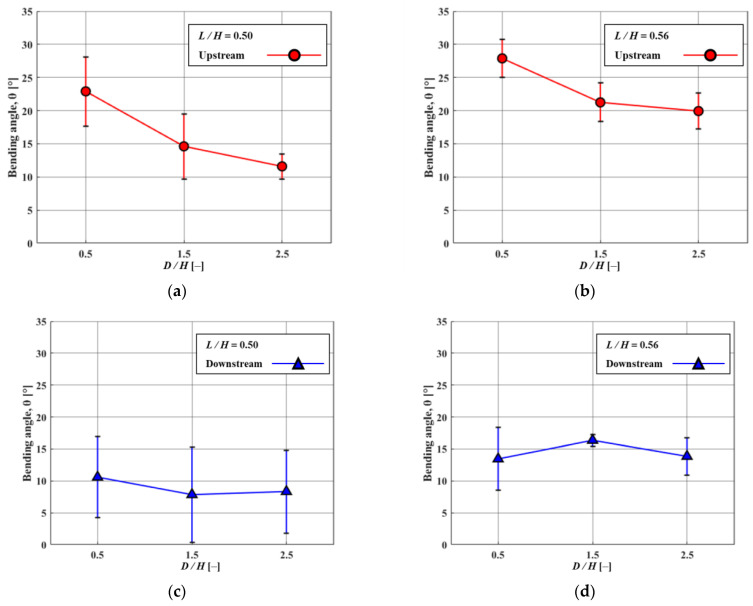
Variations in bending angles of upstream (circle) and downstream (triangle) FSs. (**a**) Upstream FS when *L/H* = 0.5. (**b**) Upstream FS when *L*/*H* = 0.56. (**c**) Downstream FS when *L*/*H* = 0.5. (**d**) Downstream FS when *L/H* = 0.56.

Figure 6 and Figure 7 show how the fluid flows in the horizontal and vorticity patterns with time corresponding to the height of FSs. Based on the experimental Section 3.1, the *D/H* was kept at 1.5 and the Re value of 3440. For the case of *L/H* = 0.56, flow reattachment was seen within the range of 1 < *D/H* < 1.5. The recirculation occurred under the attachment region in FSs. As a result, a small secondary vortex developed around the bottom of the upstream FS. This showed that particles from upstream are concentrated behind the upstream FS. Thus, when particles accumulate in a certain area, they can be effectively removed. This process can be performed using certain mechanisms such as suction devices or by using a regular cleaning operation. The temporary changes in horizontal velocity profiles with vertical position *y/H* at *L/H* = 0.56 are shown in Figure 8. The *x/D* is 80% positions of *D/H*. The magnitude of the negative velocity and temporal variations of negative velocity exhibited more at *L/H* = 0.56 compared to *L/H* at 0.5. Because the negative velocity represents the recirculation, the variation of recirculation was larger for the *L/H* = 0.56 case. The change in *L/H* of FSs has a significant impact on the negative velocity profile shift. In the present study, the removal of the particles was found to be dependent on the presence of a small secondary vortex motion. When a small secondary vortex is formed on the upstream FS, it collects particles from the tap water, particularly microplastics. Both *L/H* ratios (i.e., 0.5 and 0.56) exhibit similar vortex motions. However, in terms of pressure drop and material cost, the *L/H* ratio of 0.5 is shown to be more effective for collecting microplastics. Lower blockage ratios (*L/H* = 0.5) result in a low-pressure drop which allows water to pass through smoothly and enhances the microplastic particle collection efficiency. A comparison of various bending angles at different *L/H* ratios of 0.5 and 0.56 is presented in Figure 9. The bending angle for *L/H* at 0.56 was found to be larger compared to *L/H* at 0.5 because of the higher height of FSs. It is shown that in both cases, the bending angles of the upstream FS were larger than that of the downstream FS because the bending angles of upstream FS are influenced by the force induced by fluid momentum. On the other hand, the movement of downstream FS was influenced by the reattachment or vortex shedding generated by upstream FS. When the *D/H* increased, the bending angle of upstream FS exhibited a decreasing tendency for both *L/H* cases. However, the tendency of bending angle downstream behaved differently for both *L/H* ratios. This indicates that the separated flow from upstream structures has a significant impact on the bending angles of downstream structures. At *L/H* = 0.5, the variation of the bending angle was significantly higher. The present result could further aid in the research fields of energy harvesting.

### 3.3. Secondary Vortex Stability

**Figure 10:**
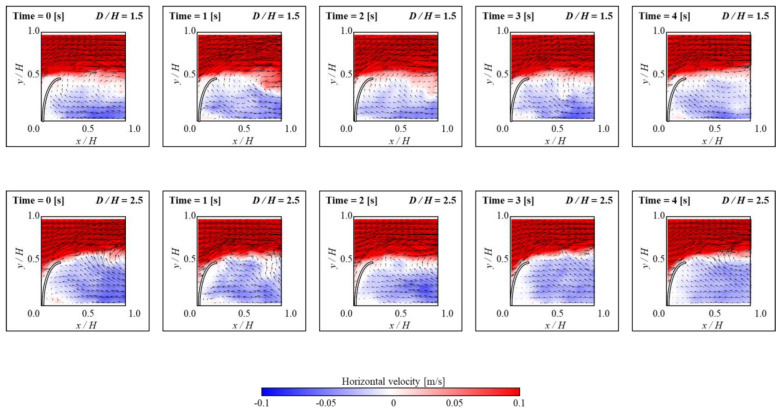
Instantaneous velocity vectors and horizontal velocity (*u*) contours of flow near the upstream FS for *D/H* = 1.5 (**upper row**), *D/H* = 2.5 (**lower row**) with an increase in time.

In the upstream FS, maintaining a small secondary vortex structure is crucial for optimal conditions of collected microplastics. Therefore, in the present study, the small secondary vortex structure near upstream FS was maintained during the experiment. Figure 3 depicts the formation of a small secondary vortex for *D/H* at both 1.5 and 2.5, respectively. In Figure 10, the contours and arrows depict how fluid velocity moves and varies over time near the upstream FS. These contours provide substantial information on horizontal velocity and velocity vector distribution, as well as flow patterns. When *D/H* is 1.5, this small secondary vortex remains unchanged. In contrast, this secondary vortex progressively dissipates when *D/H* is 2.5, as seen in the figure. As the cycle occurs multiple times within an experiment, it is anticipated that the secondary vortex exists continuously at a *D/H* of 1.5.

During an experiment, a large recirculation flow covered the gap region, leading to the dispersion of a small secondary vortex structure. Due to the absence of this small secondary vortex, microplastic dissipated behind the upstream flow. The presence of the small secondary vortex behind the upstream FS is determined by quantitative analysis. The particle collection is favorable when a small secondary vortex continues to form behind the upstream FS. The circulation (Equation (4)) is used to evaluate the persistency of the small secondary vortex structure behind the FSs, which is expressed as [40]

(4) Γ=∬ω dA

where *ω* is the vorticity vector and *dA* is the unit area. The integration area was determined where the small secondary vortex structure was shown, and the corresponding area is 18.7 mm^2^. The vorticity value was substituted into the equation (Equation (4)) to calculate the circulation value.

**Figure 11:**
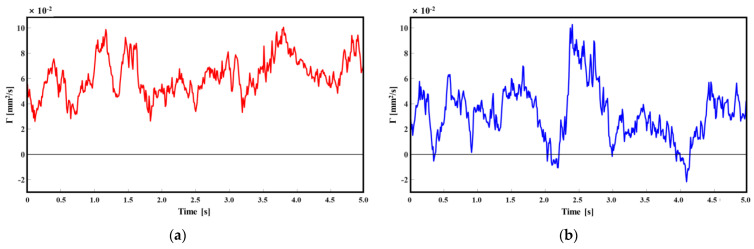
Temporal variations of circulation when (**a**) *D*/*H* = 1.5,(**b**) *D/H* = 2.5.

In Figure 11a, for *D*/*H* = 1.5, the circulation value (*Γ*) remained positive, which indicated that the vortex continuously formed in a counterclockwise direction and maintained uniformity throughout the experiment. However, at *D*/*H* = 2.5, the *Γ* value was found to be inconsistent and sometimes appeared negative, which is shown in Figure 11b. It demonstrated that the small secondary vortex structure in a counterclockwise direction could not be maintained. This implies the disappearance or rotation of the generated vortices in the opposite direction. At *D*/*H* = 2.5, several vortex shedding were observed within the 5 s experiments, therefore this particular time was maintained during the experiment. The frequency of vortex shedding led the vortex to separate from the recirculation zone and resulted in the recirculation flow moving in the x = 0 position. On the other hand, when *D*/*H* = 1.5, the large recirculation is associated with the reattachment and, as a result, the recirculation movement did not appear. Therefore, the small secondary vortex structure could be maintained. Despite the small secondary vortex structure becoming smaller due to changes in recirculation size, it did not disappear. Based on these results, *D*/*H* = 1.5 exhibits better particle collection than *D*/*H* = 2.5. Furthermore, these results appear to be persistent beyond the 5 s timeframe.

Before conducting an experiment using actual water pipes, an experimental model was designed to replicate flow conditions similar to water pipes. The objective was to determine whether FSs could effectively perform the role of removing microplastics from water pipelines. To measure similarly to a water pipe, tap water was used as a working fluid and 44 μm hollow glass particles were used. Although this experimental work was not conducted directly within water pipes through which fluid-containing microplastic flows, efforts were made to replicate actual conditions. Based on the experiment, it was confirmed that microparticles aggregated in the small secondary vortex structure behind upstream FS. Therefore, the present study suggests that installing FSs in water pipes may be a promising way to remove microplastics.

## 4. Conclusions

In this study, tandem FSs are proposed and studied experimentally for capturing microplastics. To demonstrate the efficiency of microplastic collection, the flow behaviors were compared by varying the height and spacing between the tandem FSs. The study also examined the ideal parameters that govern the efficient capture of microplastics, especially focused on the presence of a small secondary vortex structure behind the upstream FS. These optimal conditions offer significant findings that aid in the detection of microplastic using tandem FSs. At a *D/H* ratio of 0.5, the flow pattern was found to be insufficient to detect within the FSs region. However, when the *D/H* ratio increased, the flow structure developed, which in turn formed a large recirculation. Despite the presence of a large recirculation, small secondary vortex structures were consistently generated near the bottom of the upstream FS. However, when the *D/H* ratio is 2.5, vortex shedding originated and thus it contributed to the movement of the large recirculation within the FS regions. As a result, the movement of a large recirculation affected the small secondary vortex, which may potentially impact microplastic dissipation. Similar behavior is observed when the height of FSs is increased. In addition, the persistency of a small secondary vortex structure is determined based on circulation value. Unlike the *D*/*H* = 2.5 case, the secondary vortex structure is maintained at *D*/*H* = 1.5 due to positive circulation values during the experiment. The present study may be beneficial for researchers working on flow behaviors in tandem arrangements, particularly in FSs. The present results can be further used for validation, especially in pilot scale-up for the efficient capturing of microplastic particles from household tap water pipes.

## Data Availability

Data is contained within the article and Supplementary Materials.

## Supplementary Materials

The following experimental data’s used in the present figures can be downloaded at: https://www.mdpi.com/article/10.3390/mi15010100/s1.
